# Effects of Stone-Made Flour Bread and Roller Flour Bread on the Soundness of the Teeth and Nutrition Generally

**Published:** 1913-11-15

**Authors:** Thomas G. Read


### EFFECTS OF STONE-MADE FLOUR BREAD AND ROLLER FLOUR BREAD ON THE SOUNDNESS OF THE TEETH AND NUTRITION GENERALLY.

BY THOMAS G. READ, L.D.S., ENG., D.M.D., HARVARD.

From the comments of the *Miller*, published in News and Notes of the *Dental Record* for September*, it appears the writer doubts the harm done to the teeth and nutrition generally by eating bread made from roller flour.

* The following is the note referred to:—" Commenting on the assertion of Mr. Thos. G. Read in the DENTAL RECORD (page 446) that on account of eating bread made from roller flour the teeth and physique of the Jersey people are not so fine as that of Normans and Bretons, descended from the same stock, the *Miller* says 'it would be interesting to know for how many generations the writer of the above believes the Jersey people have been eating roller flour. And we rather fancy that there is plenty of roller-made flour to be found in Normandy and Brittany. If not, our milling engineers have a fine field open to them not far from home.' "

Let the *Miller* have bread composed of stone-made flour containing the unimpaired enzymes of the grain, and bread made from roller flour tested for acidity before and after mastication by an analytical chemist. The latter will report that the stone-made flour bread does not form lactic acid during mastication, and that such bread has to remain in the mouth for hours before any lactic acid is formed from it; whereas the roller flour bread does form lactic acid during mastication, and this formation of acid continues for hours when any such bread remains in the mouth. Thus the former bread has to remain in the mouth a long time before it can do any harm to the teeth by the acid combining with the lime salts of the enamel, and any crumbs lodging may be removed during the next meal before any lactic acid is formed, whereas the latter bread may quickly do harm.

To demonstrate the effects on nutrition generally of the presence and the absence of the enzymes of the grain, feed from the shell some chickens on a mixture of say, one-third sharps and two-thirds ground oats, containing the unimpaired enzymes. Mix the meal with water of a temperature of not above 100 degrees Fahrenheit, and allow the food to stand sufficient time before feeding for the enzymes to perform their actions. These chickens will develop into fine birds.

Feed other chickens on similar meal mixed with boiling water. These will not make such fine birds as the former, as the heat of the boiling water will destroy some of the enzymes.

Feed other chickens from the shell on similar meal baked, to destroy the enzymes, before mixing with water for feeding. These chickens will soon all die.

Feed other chickens alternately for a few days on the first mentioned food, and for a few days on the last mentioned. The birds that are raised on this alternating diet will be of inferior size, physique and constitution.

The above statements relating to feeding chickens are founded on the results of experiments I conducted while residing at Heathfield, Sussex, from the autumn of 1906 until the late summer of 1909.

Each breeding season I reared a large number of chickens. Non-enzymic feeding experiments with chickens need not extend over a fortnight, unless access is obtained to grass, other vegetation or any enzymic food, or they have been allowed to fill their crops well with enzymic food to commence with.

Young mammals therefore do not give such quick results. Such experiments with chickens will show the *Miller* that it matters not how many generations of Jersey people have eaten bread made from roller flour. What effects them is what they eat themselves not what their parents have eaten.

In my opinion the origin of life is the actions of the enzymes of foods, because if there were no enzymes there would be no life. A chicken is produced from an egg after it has, under certain conditions, been at a regular temperature for fixed periods. Previous to the exit of the chicken from the shell yolk of the egg is drawn into the abdomen through the navel. The bird does not require any food until about twenty-four hours after hatching. The yolk of egg contains enzymes. If the chicken is fed on a non-enzymic diet it will go on all right for a few days; this is apparently due to the enzymes of the yolk. After about four days if it does not receive some enzymic food it will rapidly go wrong and soon die. If a chicken be fed for the first few days on an enzymic diet, until it fills its crop well, and then be placed on an non-enzymic diet the period before it goes wrong is considerably increased.

The young are more quickly affected by non-enzymic food than adults, apparently owing to the younger the life the lower the vitality.

Broodies should be well fed with the same food as the chickens. If well fed on enzymic food they usually lay before being removed from the chickens. If a hen which is brooding chickens be fed for about a fortnight on non-enzymic food, she takes no apparent harm, but usually she then does not lay until she has moulted, and then is in no hurry to restart laying.

Laying hens, when fed on corn which has been baked to destroy the enzymes, and not allowed access to any enzymic food, soon stop laying.

During the rearing season of 1907 a number of broods of chickens were fed by me on non-enzymic food obtained by baking Sussex ground oats and sharps, similar to the Sussex ground oats and sharps unbaked which the other birds were being fed on. Boiling water was poured into a pail or a basin, and when the temperature had fallen to 100 degrees Fahrenheit, one part of sharps was mixed with the water, then four parts of ground oats were added and the mass left for sometime. Before feeding another one part of sharps was mixed in. The object of using any sharps is that ground oats are inclined to stick to the beaks of the birds. The broods fed on this non-enzymic food were kept on a bare roadway, in coops with runs in front. Chickens placed on this diet were all dead in about a fortnight. Other chickens did well fed on similar meal, except it was not baked before mixing with water in a similar manner, and thus during cooling the cellulase rendering the cellulose soluble and the diastase or amylase converting starch into sugar. One part of unimpaired diastase is potent enough to convert two thousand parts of starch into sugar. A few broods were fed on broken oat cake, made by mixing two-thirds of Sussex ground oats and one-third of sharps with tepid water and then baking it. In this cake the enzymes allulase and diastase performed their actions until the dough reached a temperature of above 100 degrees Fahrenheit. The chickens fed on this cake did equally well. In 1908 the most remarkable features of the experiments were some chickens reared on an alternating enzymic and non-enzymic diet. Some died during these experiments, and those reared were not birds to be proud of. When the surviving chickens were removed from the broodies they were placed on an enzymic diet.

Most of these chickens went into fattening crates except a few pullets and cockerels, which were retained for further research. In 1908 a fattener, who had had birds from me the previous year remarked that the birds he was then having from me were some of them not such good ones for the crate as those of the first year. This he thought was due to the birds of the previous year having been reared on new ground. It was not mentioned to him that some had been differently fed.

In the winter of 1908 some of the pullets reared on the alternating diet were mated with an unrelated male bird. These pullets proved to be poor layers; when their eggs were fertile the germs were mostly weak, and any chickens hatched did badly, although fed on an enzymic diet. Breeding from the pullets reared on the alternating diet was soon discontinued.

For an example of the marvelous recuperative action of enzymic food, see how quickly a stale hunter or other horse regains his vigour when turned out to grass.

The stone-made flour bread eating Bretons, who early in the year sail from St. Brieuc for St. Helier to dig and plant the Jersey potato lands, show that dental caries is due more to local causes rather than any physiological one, because if they stay too long in Jersey they return to Brittany with decayed teeth, instead of the sound ones they came with. This cannot be due to any faulty development of the actual tooth-fabric, because these Bretons, while in Brittany eating the stone-made flour bread of the country, are very free from carious teeth, and it is these same teeth that start to decay while in Jersey eating bread made from imported roller flour. The value of the flour imported is about £72,000 annually. Jersey does not produce 850 acres of wheat and imports none.

My attention was first drawn to the Breton teeth decaying in Jersey when residing in St. Brelade's Bay. On visiting a farm from which I obtained eggs, I noticed a very fine Breton with very bad teeth. On remarking what bad teeth he had, he said:—"Oh, my teeth were all right until I had lived some time in this island." On enquiring into the matter I found it was a recognized thing for Breton laborers to have carious teeth after they had lived about two years in Jersey. No one seemed to regard it as of much importance, as the Jersey people have such carious teeth. St. Helier has a population of 27,000, there are ten dentists, all at different addresses, and Jersey has thirty-five medical practitioners and a population of 52,000. Jersey, for the welfare of her own people, should produce flour in her own stone mills.

The United Kingdom, with a cultivated area of 47,001,961 acres, produces about 2,000,000 acres of wheat, and 60,000 acres of rye.

France with a cultivated area of 85,758,776 acres, produces about 16,000,000 acres of wheat and 3,000,000 acres of rye. That is, France is practically self-supporting in the matter of bread stuffs.

France has quarries from which are obtained the best mill-stones for grinding grain.

French wheats, like English wheats, are soft and are best ground between stones. Thus there is not such a fine field in Normandy and Brittany for our roller-milling engineers as the *Miller* would suggest.

The French Government, knowing that the more wheat-offals produced in the country the greater the number of head of stock kept, permits millers, if they export a certain amount of flour, to import duty free a certain amount of wheat, although there is a French import duty of 12s. 3d. per quarter on wheat. To import wheat duty free, the percentage of flour required to be exported is well below the amount of flour that can be obtained from the imported wheat. Thus, when the price of imported wheat is well below the French price, a good profit can be obtained by exporting flour. In France the price of wheat is usually about six shillings more than in the United Kingdom.

Nearly all the wheats that are exported from different parts of the world, are hard wheats, and since hard wheats are best suited for roller milling, the above system of admitting wheat duty free, when flour is exported, has encouraged roller milling at certain French ports. Some of the roller flour produced in France is used in making pastry, and some for mixing with the stone-made flour of the country for bread-making. The amount of bread made in France solely from roller flour is not great, as there is a taste there for flavor in the loaf and that cannot be produced unless the unimpaired enzymes of the grain are in the flour. This is why French bread is so nice.

At the recent International Medical Congress three papers were read that I believe will prove to relate to the importance of the enzymes of foods on nutrition generally.

Dr. E. H. Beall, of Texas, read a paper on "The Invasion of America by Pellagra." He stated there are 30,000 pellagrins in the United States, and that last year 3,000 persons died there from pellagra. He mentioned pellagra has been known in Italy for 200 years.

I would point out that macaroni, a non-enzymic food, is largely consumed in Italy.

Dr. Charles E. Stewart, of Battle Creek, Michigan, read a paper on the probable identity of Pellagra and Spure. He pointed out that the diet which gave the best results in cases of sprue, namely, milk and strawberries, also gave equally good results in cases of pellagra.

I suggest it would be difficult to name an enzymic diet in which the enzymes are more active than in new milk and fresh strawberries.

Dr. H. Schaumann, of Hamburg, read a paper on "Pathological conditions due to defects in diets." He pointed out that complete nutrition did not depend only on the presence in the food of a sufficient amount of the main nutritive substances, namely, albumin, carbohydrates, fat, mineral compounds and water. The presence in the food of certain stuffs, which were unknown and consequently neglected formerly, were of just as much importance for the maintenance of the organism in the higher animals.

I believe the reason why these certain stuffs are stated to be unknown is that a substance or substances have been sought, while I consider the important factors for the maintenance of good health are actions and these actions are those performed by the enzymes of the foods, which develop the full nutritive quantities of the foods they are contained in.

I regard dental caries, pellagra, sprue, beri-beri and many of the mysterious diseases at present afflicting the human race, and in some cases domestic stock, as due to non-enzymic foods; and except dental caries, the differing phenomena developed as due to other causes lowering the vitality of the system.

When the truth of the effects on nutrition of the impairment, destruction or elimination of the enzymes of foods is established, non-enzymic bread made from roller-flour will soon be a mistake of the past.—*Dental Record.*

